# Supplementary material for: Content-rich biological network constructed by mining PubMed abstracts
Source: BMC Bioinformatics. 2004 Oct 8;5:147. doi: 10.1186/1471-2105-5-147 (PMC528731; doi:10.1186/1471-2105-5-147)
Supplement: Additional File 2 — The original results of the above study (non-essential files are deleted to keep the file size under the limit set by BMC bioinformatics). [file 1471-2105-5-147-S2.bz2 › chilibotAdditionalFile2/dip10/616ID10360578E11000/html/CDC42_WAS.html]

 


 **CDC42** and **WAS** 
  
Found 126 abstracts in PubMed, retrieved 10.  
 

 What does Google say? 
 PDF only 
| .edu only 

---

**Interactive relationship** (e.g. stimulation, inhibition, etc)

**Neutral relationship**- In Vav 1 deficient T cells, WASP  [ **WAS** ]  recruitment proceeds normally, but localized activation of  **Cdc42**  and WASP  [ **WAS** ]  is disrupted.  Ref: 12874226 J ImmunolJ Immunol,
- SLP 76 Coordinates Nck Dependent Wiskott Aldrich Syndrome Protein  [ **WAS** ]  Recruitment with Vav 1  **Cdc42**  Dependent Wiskott Aldrich Syndrome Protein  [ **WAS** ]  Activation at the T Cell APC Contact Site.  Ref: 12874226 J ImmunolJ Immunol,
- We have shown that WIP interacts with members of the Wiskott Aldrich syndrome protein  [ **WAS** ]  family and is essential for filopodium formation regulated by  **Cdc42**  GTPase.  Ref: 12724353 J Cell Sci, 2003
- We show that the endocytic protein, dynamin, competes for binding to the SH3 domains with the neural Wiskott Aldrich Syndrome protein  [ **WAS** ] , an actin filament nucleation protein that is a substrate for activated  **Cdc42** .  Ref: 12686614 Mol Biol Cell, 2003
- These discoveries have fuelled interest in understanding both the role of the actin cytoskeleton as an integral component of lymphocyte activation and the interplay between lymphoid cell cell contact sites immunological synapse, retractile pole structures uropod, distal pole complex, and Rho family GTPases Rac, Rho,  **Cdc42** , their upstream activators Dbl family guanine nucleotide exchange factors and their downstream effectors WASp  [ **WAS** ] , Arp2 3, ADAP.  Ref: 12787750 Curr Opin Immunol, 2003
- The Wiskott Aldrich syndrome protein  [ **WAS** ]  WASP  [ **WAS** ]  is an effector of the Rho family GTPase  **Cdc42** , whose activation leads to stimulation of the actin nucleating assembly, Arp2 3 complex.  Ref: 12769846 Mol Cell, 2003

**Non-interactive relationship** (e.g. studied together, co-existance, homology, etc.)

- The Rho protein  **Cdc42**  induces a significant conformational change in its downstream effector, the Wiskott Aldrich syndrome protein  [ **WAS** ]  WASP  [ **WAS** ] .  Ref: 12680752 Biochemistry, 2003
- Active  **Cdc42**  and N WASP  [ **WAS** ]  are recruited to exocytosing cortical granule membranes before F actin coat assembly and coats assemble by  **Cdc42**  dependent, de novo actin polymerization.  Ref: 12872130 Nat Cell BiolNat Cell Biol, 2003
- N WASP  [ **WAS** ]  localizes at the cell periphery, where it controls actin polymerization downstream of signal molecules such as adapter proteins,  **Cdc42** , Src family kinases, and phosphoinositides.  Ref: 12871950 J Biol ChemJ Biol Chem, 2003
- The recruitment and activation of WASP  [ **WAS** ]  are coordinated by tyrosine phosphorylated Src homology 2 domain containing leukocyte protein of 76 kDa, which functions as a scaffold, bringing Nck and WASP  [ **WAS** ]  into proximity with Vav 1 and  **Cdc42**  GTP.  Ref: 12874226 J ImmunolJ Immunol, 2003
- We demonstrate that efficient phosphorylation and dephosphorylation of WASP  [ **WAS** ]  at Y291 are both contingent on binding to activated  **Cdc42** .  Ref: 12769846 Mol Cell, 2003
- We have shown previously that Wiskott Aldrich syndrome protein  [ **WAS** ]  WASP  [ **WAS** ]  activation at the site of T cell APC interaction is a two step process, with recruitment dependent on the proline rich domain and activation dependent on binding of  **Cdc42**  GTP to the GTPase binding domain.  Ref: 12874226 J ImmunolJ Immunol, 2003
- SMC induced matrix contraction  **was**  markedly blocked 80% by inhibiting the Rho p160ROCK pathway and myosin light chain kinase, and  **was**  decreased to a lesser extent 30 40% by a negative mutant of Rac and inhibitors of phosphatidylinositol 3 kinase PI 3 kinase or p38 mitogen activated protein kinase MAPK, but it  **was**  not affected by the inhibition of Ras and  **Cdc42**  Wiskott Aldrich syndrome protein  [ **WAS** ]  WASP  [ **WAS** ]  pathways.  Ref: 12891007 J Vasc ResJ Vasc Res, 2003
